# Genomic characterization of a mobile-element-associated resistance island in trimethoprim-sulfamethoxazole-resistant *Stenotrophomonas maltophilia* isolates sharing the same PFGE genotype

**DOI:** 10.3389/fcimb.2026.1926102

**Published:** 2026-09-11

**Authors:** Gülşah Altan, Erva Rakıcı, Devrim Dündar, Osman Birol Özgümüş

**Affiliations:** 1 Department of Medical Microbiology, Faculty of Medicine, Kocaeli University, Kocaeli, Türkiye; 2 Department of Medical Microbiology, Faculty of Medicine, Recep Tayyip Erdoğan University, Rize, Türkiye

**Keywords:** antimicrobial resistance, mobile genetic elements, molecular epidemiology, pulsed-field gel electrophoresis, resistance island, *Stenotrophomonas maltophilia*, whole-genome sequencing

## Abstract

**Background:**

*Stenotrophomonas maltophilia* is an opportunistic nosocomial pathogen with intrinsic multidrug resistance and limited therapeutic options. This study integrated phenotypic susceptibility testing, targeted molecular screening, PFGE and short-read whole-genome sequencing (WGS) to characterize resistance determinants and their local genomic context in clinical-culture isolates.

**Methods:**

Thirty-four non-duplicate clinical-culture isolates were analyzed. Susceptibility to trimethoprim-sulfamethoxazole (TMP-SMX), levofloxacin and minocycline was determined by disk diffusion. Minocycline testing was repeated with acceptable quality control, and the repeat-run zone records were reassessed using current CLSI breakpoints. A predefined, hypothesis-driven PCR panel was used to screen selected resistance-, efflux- and biofilm-associated targets, and *gyrA*, *gyrB*, *parC* and *parE* were sequenced. Genetic relatedness was assessed by *Xba*I-PFGE. Three selected isolates (S05, S08 and S25) underwent WGS as a targeted genomic-context analysis rather than a whole-collection population-genomic study.

**Results:**

Resistance rates were 5.9% for TMP-SMX, 8.8% for levofloxacin and 11.8% for minocycline after reassessment using current CLSI minocycline breakpoints. *Smqnr*- and *sul1*-target PCR positivity was observed in 61.8% and 8.8% of isolates, respectively. A *dfrA27*-target PCR amplicon was observed in 82.4% of isolates but was not associated with TMP-SMX resistance and was not confirmed in the three sequenced genomes. No QRDR hotspot mutations were identified. The two TMP-SMX-resistant isolates, S05 and S08, shared the same PFGE genotype. Short-read WGS identified an identical 19,361-bp mobile-element-associated resistance region in these isolates, containing a putative Sul-family/*folP*-like dihydropteroate synthase (DHPS) annotation, two assembly-predicted hits each of *bla_OXA-2_* and *ant(3’’)*, multiple insertion sequences and a mercury-resistance module; the region was absent from S25.

**Conclusions:**

The identical resistance region in two TMP-SMX-resistant isolates sharing the same PFGE genotype is compatible with shared ancestry, vertical inheritance or persistence of an island-carrying PFGE-defined genotype; horizontal transfer was not demonstrated. The discordance between *dfrA27*-target PCR positivity, phenotype and WGS highlights the need to interpret molecular screening together with phenotypic susceptibility and orthogonal confirmation. The WGS component provides a local genomic-context characterization rather than evidence of cross-genotype dissemination.

## Graphical Abstract

**Figure:**
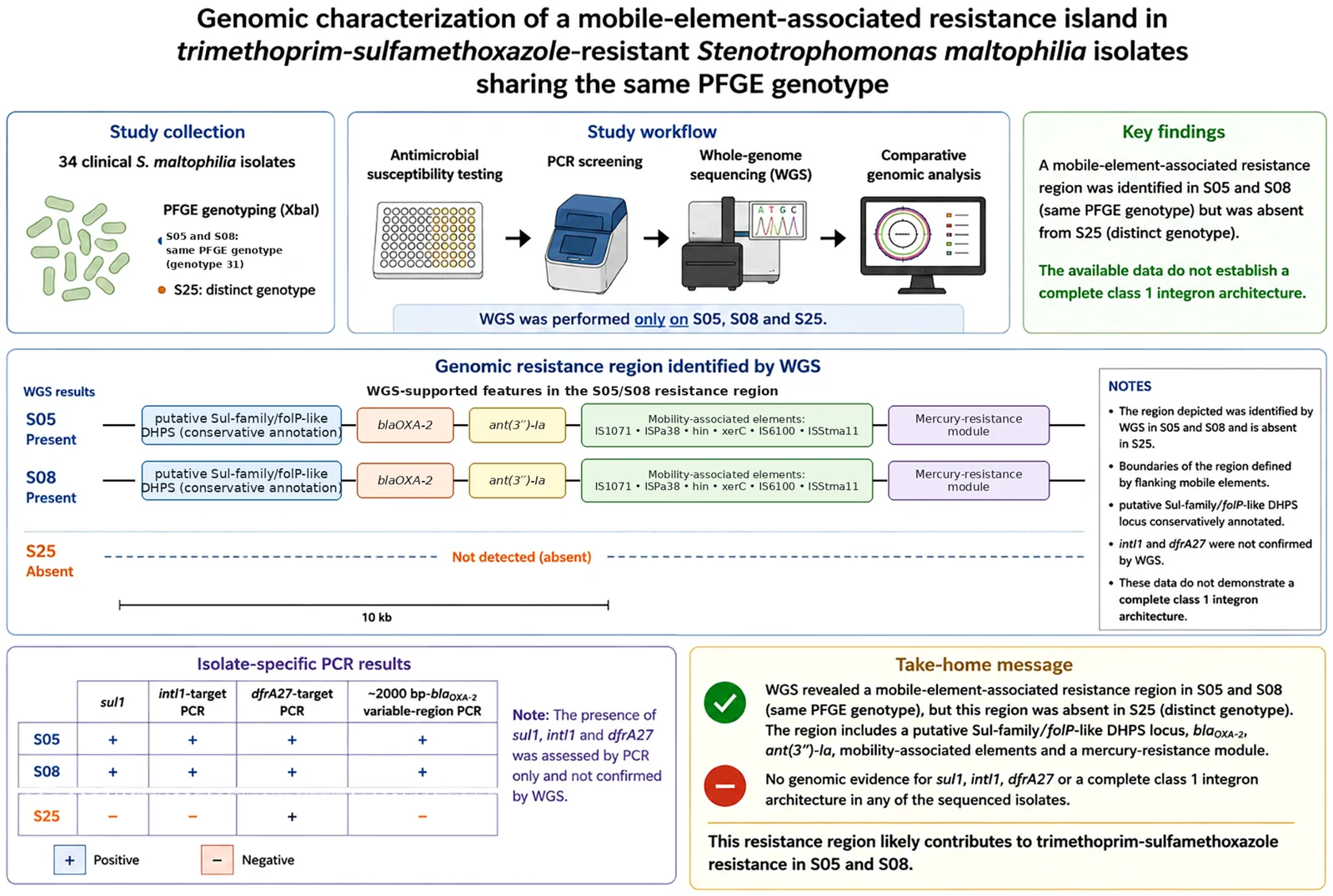

## Introduction

*Stenotrophomonas maltophilia* is an opportunistic, non-fermenting Gram-negative bacterium that has emerged as an important nosocomial pathogen, particularly among immunocompromised patients and individuals exposed to prolonged hospitalization, broad-spectrum antimicrobial therapy or invasive procedures. The organism is frequently associated with respiratory tract infections, bloodstream infections, wound infections and device-related infections, especially in intensive-care and other high-risk hospital settings (Flores-Treviño et al., 2019).

One of the major clinical concerns regarding *S. maltophilia* is its intrinsic and acquired resistance to multiple antimicrobial classes. This resistance is mediated by a combination of chromosomally encoded β-lactamases, multidrug efflux systems, reduced outer-membrane permeability and biofilm-forming capacity (Flores-Treviño et al., 2019; Zając et al., 2022). In addition, acquisition or carriage of mobile resistance determinants, including integron-associated gene cassettes, sulfonamide resistance genes and dihydrofolate reductase variants, may further complicate treatment options (Hu et al., 2011).

Trimethoprim-sulfamethoxazole remains one of the principal therapeutic agents for *S. maltophilia* infections; however, resistance mediated by sulfonamide resistance genes and trimethoprim resistance determinants has increasingly been reported (Hu et al., 2011). Fluoroquinolones and tetracycline derivatives are commonly considered alternative treatment options, but resistance mechanisms involving chromosomal quinolone-protection determinants such as *Smqnr*, efflux systems and regulatory changes may reduce their effectiveness (Shimizu et al., 2008; Zając et al., 2022).

Mutations in quinolone resistance-determining regions (QRDRs) of topoisomerase genes, particularly *gyrA*, *gyrB*, *parC* and *parE*, have been investigated in *S. maltophilia*. However, their contribution to fluoroquinolone resistance appears to be less consistent than in several other Gram-negative pathogens, suggesting that non-QRDR mechanisms, including efflux-mediated resistance and intrinsic protection systems, may play a more prominent role in this species (Valdezate et al., 2002; Jia et al., 2015; Ribera et al., 2002).

Beyond antimicrobial resistance, biofilm formation and efflux pump systems are important contributors to persistence, environmental survival and colonization capacity of *S. maltophilia* in healthcare settings (Nicoletti et al., 2011; Zhuo et al., 2014; Flores-Treviño et al., 2019; Zając et al., 2022). These traits may facilitate survival on hospital surfaces and medical devices, promoting sporadic infections, device-associated colonization and occasional outbreaks.

Although sul genes and integron-associated TMP-SMX resistance have been reported previously, fewer studies have integrated phenotypic susceptibility, targeted PCR/Sanger analysis, PFGE and WGS to examine both the local genomic organization of resistance determinants and discordance between molecular screening and phenotype wit*hin* the same clinical-culture collection.

Therefore, the present study aimed to investigate the antimicrobial susceptibility patterns, selected resistance-associated determinants, efflux- and biofilm-related markers, QRDR sequence profiles and PFGE-based genetic relationships of clinical-culture *S. maltophilia* isolates recovered from a tertiary-care hospital. In addition, three selected isolates were subjected to short-read WGS to characterize the local genomic context of resistance-associated determinants. This targeted WGS component was not designed to establish whole-collection population structure, cross-genotype distribution, element origin or functional gene expression.

## Materials and methods

### Study design, bacterial isolates and species identification

This study included 34 non-duplicate clinical-culture *Stenotrophomonas maltophilia* isolates recovered from routine diagnostic cultures between February 2022 and January 2024 at the Central Laboratory Bacteriology Unit of Kocaeli University Research and Application Hospital, Türkiye. The isolates represented different specimen types, including urine, wound, sputum, catheter, tracheal aspirate, cerebrospinal fluid and pleural fluid samples. Only one isolate per patient/sample episode was included. Species-level identification was performed using a MALDI-TOF MS system (bioMérieux, France) according to the manufacturer’s instructions. Isolates identified as *S. maltophilia* were stored at −80 °C in beaded bacterial storage tubes (RTA, Türkiye) until further molecular analyses. The study was conducted using anonymized bacterial isolates and did not involve patient-identifiable data. Ethical approval was obtained from the Kocaeli University Faculty of Medicine Non-Interventional Clinical Research Ethics Committee on 1 February 2024 with approval number GOKAEK-2024/02.41 and project number 2024/65.

### Antimicrobial susceptibility testing

Antimicrobial susceptibility testing was performed in January 2024 by the disk diffusion method on Mueller–Hinton agar according to Clinical and Laboratory Standards Institute (CLSI) recommendations. The antimicrobial agents tested were trimethoprim-sulfamethoxazole (TMP-SMX; 1.25/23.75 µg), levofloxacin (LEV; 5 µg), and minocycline (MIN; 30 µg). At the time of testing, categorical results were interpreted using CLSI M100, 30th edition (2020) (Clinical and Laboratory Standards Institute, 2020), which was the laboratory reference document in routine use. Quality-control testing used *Escherichia coli* ATCC 25922. The final QC zone diameters were 30 mm for LEV, 28 mm for TMP-SMX, and 25 mm for the repeat MIN run. The repeat MIN run, for which the ATCC 25922 QC result was wit*hin* the applicable range, was used for the final minocycline analysis. To address subsequent breakpoint revisions, these repeat-run MIN zone records were retrospectively reassessed using the current CLSI criteria for *S. maltophilia* (30-µg disk: susceptible ≥26 mm, intermediate 21–25 mm, resistant ≤20 mm), which were first introduced in M100 Ed34 and retained in current editions (Clinical and Laboratory Standards Institute, 2025; Clinical and Laboratory Standards Institute, 2026). Exact MIN zone diameters were recorded for 23 isolates; for 11 isolates the repeat-run worksheet retained the notation “>20 mm,” which establishes non-resistance under the current breakpoint but does not permit retrospective differentiation between intermediate and susceptible categories. For categorical LEV or TMP-SMX results recorded as resistant without a numerical diameter, the contemporaneous worksheet indicated no visible inhibition zone (growth up to the disk edge); no numerical zone diameter was retrospectively assigned.

### Genomic DNA extraction

Total bacterial DNA was extracted from pure overnight cultures using the phenol-chloroform-isoamyl alcohol method as previously described by Chen and Kuo (1993), with minor laboratory modifications. DNA extracts were stored at −20 °C until use in PCR assays and sequencing analyses.

### PCR screening of resistance-associated, integron, efflux and biofilm-related genes

PCR assays were performed using a predefined, hypothesis-driven panel based on resistance, efflux and biofilm determinants previously reported in *S. maltophilia*. The tested targets included class 1, class 2 and class 3 integrase genes (*intI1*, *intI2* and *intI3*), class 1 and class 2 integron variable regions, sulfonamide resistance genes (*sul1* and *sul2*), quinolone-associated determinants (*Smqnr*, *qnrA*, *qnrB*, *qnrC*, *qnrD*, *qnrS*, *aac(6′)-Ib-cr* and *qepA*), β-lactamase genes (*L1* and *L2*), dihydrofolate reductase genes (*dfrA1*, *dfrA5*, *dfrA12*, *dfrA13*, *dfrA17* and *dfrA27*), *oqxA* and *oqxB*, efflux-associated genes (*smeA*, *smeD*, *smeK*, *smeI*, *smeH*, *smeW* and *smrA*) and biofilm-related genes (*spgM*, *rmlA*, *smf-1* and *rpfF*). The panel was intended for targeted screening rather than exhaustive resistome, regulatory-network or operon characterization. For multigene efflux systems, selected loci were used as screening markers; detection of an individual marker does not establish completeness, expression or functional activity of the corresponding operon. The original sulfonamide-resistance panel included *sul1* and *sul2*; *sul3* and *sul4* were outside the predefined protocol. PCR reactions were performed in a final volume of 25 µL containing template DNA, gene-specific primers, reaction buffer, dNTPs and DNA polymerase. Appropriate positive and negative controls were included in each PCR run where available. Primer sequences, expected amplicon sizes and original references are provided in **Supplementary Table 1** (Lévesque et al., 1995; White et al., 2001; Machado et al., 2005; Park et al., 2006; Robicsek et al., 2006; Cattoir et al., 2007; Kim et al., 2009; Pompilio et al., 2011; Flores-Treviño et al., 2014; Kargar et al., 2014; Qiu et al., 2019). PCR cycling conditions for each target group are summarized in **Supplementary Table 2**. PCR products were separated by agarose gel electrophoresis, stained and visualized under UV illumination using a gel documentation system. Amplicon sizes were estimated by comparison with an appropriate DNA ladder. PCR positivity was defined by the presence of an amplicon of the expected size and, unless independently sequenced, was interpreted as a target-specific PCR signal/amplicon rather than definitive gene carriage. In particular, *intI1*- and *dfrA27*-target PCR signals were not independently sequence-confirmed.

### Sequencing of integron variable regions and quinolone resistance-associated genes

PCR amplicons corresponding to the class 1 integron variable regions were purified and submitted for Sanger sequencing using the same amplification primers. Sequencing was performed by Macrogen Inc. (Amsterdam, The Netherlands). The resulting sequences were compared with sequences in the GenBank database using BLAST. To investigate quinolone resistance-determining regions (QRDRs), PCR amplicons of *gyrA, gyrB, parC* and *parE* were also sequenced. The obtained sequences were aligned with the *S. maltophilia* K279a reference genome sequence (GenBank accession no. AM743169.1). The analyzed regions were inspected for amino acid substitutions in known quinolone resistance-associated hotspot regions. Isolates without hotspot changes were interpreted as wild-type or as carrying only non-QRDR polymorphisms within the analyzed regions. The isolate-level QRDR sequence-analysis summary is provided in **Supplementary Table 3**.

### Pulsed-field gel electrophoresis

Genetic relatedness among *S. maltophilia* isolates was evaluated by *Xba*I pulsed-field gel electrophoresis (PFGE) using a modified version of the optimized protocol described by Durmaz et al. (2009). Briefly, genomic DNA embedded in agarose plugs was digested with XbaI and electrophoresed using a CHEF-DR II system (Bio-Rad Laboratories, Belgium). Electrophoresis was performed at 14 °C and 6 V/cm using two consecutive switch-time blocks: 16 h with switch times ranging from 5 to 30 s, followed by 4 h with switch times ranging from 1 to 5 s. After electrophoresis, gels were stained with ethidium bromide, visualized under UV illumination, and photographed. Gel images were saved in TIFF format and analyzed using BioNumerics software version 6.01 (Applied Maths, Belgium). Banding-pattern similarities were calculated using the Dice similarity coefficient, and dendrograms were generated by the unweighted pair-group method with arithmetic mean (UPGMA). Band-position tolerance and optimization were both set at 1.0%. PFGE genotypes were assigned using an 85% similarity cutoff, and band-pattern relatedness was interpreted in accordance with the criteria proposed by Tenover et al. (1995).

### Selection of isolates for whole-genome sequencing

Three isolates were selected wit*hin* a predefined targeted WGS design to characterize the genomic context of resistance-associated determinants rather than to perform whole-collection population genomics. S05 and S08 were selected because both were resistant to TMP-SMX and shared the same PFGE genotype. S25 was included as a comparative isolate representing a distinct phenotypic and PFGE background. S07 was *sul1*-target PCR positive and carried an approximately 2000-bp *bla_OXA-2_*-containing variable-region amplicon but was TMP-SMX-susceptible; it was not included because the three-isolate design prioritized the two resistant isolates sharing the same PFGE genotype and one phenotypically and PFGE-distinct comparator. Consequently, the WGS design cannot evaluate sharing of the S05/S08 resistance region across distinct PFGE genotypes.

### Whole-genome sequencing, assembly and annotation

Genomic DNA from isolates S05, S08 and S25 was extracted from pure bacterial cultures and submitted for paired-end whole-genome sequencing as a commercial service by Macrogen Inc. (Amsterdam, The Netherlands). Libraries were prepared using a TruSeq PCR-free approach and sequenced on the Illumina NovaSeq X platform using 2 × 150-bp paired-end reads. Provider quality control used FastQC version 0.11.7 and Trimmomatic version 0.38; reads were retained when 90% of bases had Phred scores ≥20 and adapter sequences were removed. Genome-size estimation used Jellyfish version 1.1.12 and GenomeScope version 1.0. Because quality-filtered data exceeded approximately 150× of the estimated genome size, the provider randomly subsampled the filtered reads before assembly. *De novo* assembly was performed using SPAdes version 3.15.0 (Bankevich et al., 2012). Raw theoretical depth was calculated as raw bases divided by estimated genome size, filtered theoretical depth as filtered bases divided by estimated genome size, and assembly-input depth as reduced/subsampled bases divided by final assembly length. Accordingly, the approximately 149× values reported for the assemblies refer specifically to the subsampled assembly-input data, whereas raw theoretical depths were approximately 697×, 777× and 841× for S05, S08 and S25, respectively. Assembly quality was evaluated using total assembly length, contig count, N50, largest contig and GC content. Genome completeness was assessed using BUSCO version 5.1.3 with the bacteria_odb10 lineage dataset (Seppey et al., 2019). Draft annotation used Prokka version 1.14.6 (Seemann, 2014); the provider annotation workflow also included eggNOG-mapper version 2.1.10 with eggNOG database version 5.0.2 and InterProScan version 5.34-73.0. Provider mapping/comparison outputs used BWA version 0.7.17-r1188, BLAST version 2.14.0 and pyani version 0.2.7. Detailed raw, filtered and assembly-input metrics are provided in **Supplementary Table 5**.

### Genome-based analysis of antimicrobial resistance determinants and resistance-island context

Antimicrobial resistance-associated determinants in the WGS-selected isolates were screened using ABRicate version 1.0.1 against MEGARes version 3.0, with minimum identity and coverage thresholds of 80% and 70%, respectively (Doster et al., 2020). Annotated GFF and GBK files were additionally inspected manually for resistance determinants and related genomic features, including sulfonamide, β-lactam, aminoglycoside, quinolone-associated, efflux-associated, antiseptic/biocide-associated and metal-resistance determinants. The S05/S08 locus is conservatively designated as a putative Sul-family/*folP*-like dihydropteroate synthase (DHPS) annotation. ABRicate/MEGARes classified the corresponding hit as SULI (MEG_6615; 99.90% nucleotide identity; 100% coverage), whereas Prokka annotated an 840-nt CDS as *folP_1*/dihydropteroate synthase. The separate *sul1*-target PCR product was not sequence-validated and was not used as sequence confirmation of this WGS locus. The determinant sequence, database call, thresholds, coordinates and local genomic context are provided in **Supplementary Table 5**. Resistance-associated regions were extracted from annotated contigs and compared among the three WGS-selected isolates. In S05 and S08, the shared resistance-associated region was identified on contig 6 and manually curated according to gene coordinates and strand orientation; the S08 region was reverse-complement aligned to the S05 region to compare gene organization and nucleotide identity. Gene-neighborhood maps were generated to visualize resistance annotations, insertion sequence elements, recombination-associated features and the mercury-resistance module. Copy number was inferred from short-read draft assemblies and was therefore treated as assembly-predicted rather than definitive. Because *intI1* and *dfrA27* were not directly confirmed in the WGS annotation outputs of the selected isolates, the shared region was described as a mobile-element-associated resistance island rather than a confirmed class 1 integron.

### Statistical analysis

Statistical analyses were performed using IBM SPSS Statistics for Windows, version 29.0 (IBM Corp., Armonk, NY, USA). Descriptive statistics were used to summarize antimicrobial susceptibility results and the distribution of resistance-associated molecular determinants. Categorical variables were expressed as numbers and percentages. Because only two isolates were TMP-SMX-resistant and three were levofloxacin-resistant, phenotype–determinant comparisons were prespecified and analyzed as exploratory using two-sided Fisher’s exact tests. The comparisons were TMP-SMX resistance versus *sul1*-target PCR positivity, TMP-SMX resistance versus *dfrA27*-target PCR positivity, and levofloxacin resistance versus *Smqnr*-target PCR positivity. To provide an effect-size estimate suitable for the zero cells in these sparse 2 × 2 tables, Haldane–Anscombe-corrected odds ratios (ORs) were calculated by adding 0.5 to all four cells, with approximate 95% confidence intervals derived on the log-OR scale. A p value of <0.05 was used as the nominal threshold for these exploratory tests; the very wide confidence intervals were considered when interpreting the findings. QRDR results were reported descriptively because no hotspot mutation was detected in any isolate. PFGE and WGS findings were also interpreted descriptively and were not subjected to inferential statistical testing.

### Sequence data availability

The raw whole-genome sequencing reads are publicly available in the NCBI Sequence Read Archive under BioProject PRJNA1481107 (https://www.ncbi.nlm.nih.gov/bioproject/PRJNA1481107), released on 17 August 2026. Isolate-to-record mapping is as follows: S05, BioSample SAMN61058391 (https://www.ncbi.nlm.nih.gov/biosample/SAMN61058391) and SRA run SRR39278206 (https://www.ncbi.nlm.nih.gov/sra/SRR39278206); S08, BioSample SAMN61058392 (https://www.ncbi.nlm.nih.gov/biosample/SAMN61058392) and SRA run SRR39278205 (https://www.ncbi.nlm.nih.gov/sra/SRR39278205); and S25, BioSample SAMN61058393 (https://www.ncbi.nlm.nih.gov/biosample/SAMN61058393) and SRA run SRR39278204 (https://www.ncbi.nlm.nih.gov/sra/SRR39278204).

## Results

### Clinical-culture isolate collection

A total of 34 non-duplicate clinical-culture *Stenotrophomonas maltophilia* isolates recovered from routine diagnostic cultures between February 2022 and January 2024 were included in the study. The isolates were obtained from diverse specimens, most commonly sputum (12/34, 35.3%), urine (8/34, 23.5%), wound specimens (6/34, 17.6%) and catheter samples (4/34, 11.8%). Tracheal aspirate samples accounted for 2/34 isolates (5.9%), whereas cerebrospinal fluid and pleural fluid samples each accounted for 1/34 isolate (2.9%). Detailed isolate-level microbiological, phenotypic and molecular data are provided in **Supplementary Table 4**.

### Antimicrobial susceptibility profiles

**Figure 1:**
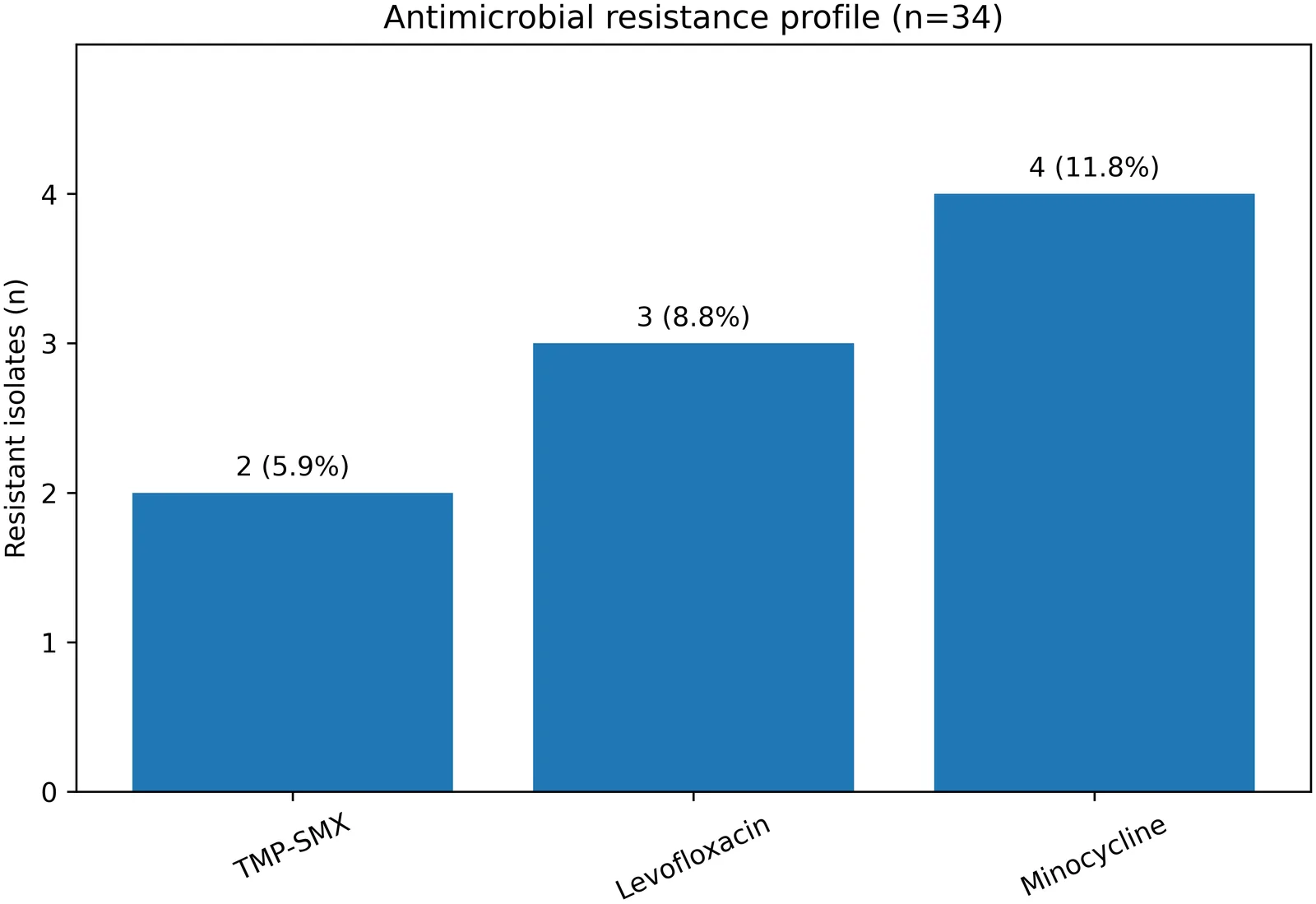
Antimicrobial resistance profile of clinical-culture *Stenotrophomonas maltophilia* isolates (n = 34). Bars represent the number of isolates resistant to trimethoprim-sulfamethoxazole (TMP-SMX), levofloxacin and minocycline. Percentages were calculated using the total number of isolates as the denominator. Resistance was detected in 2/34 (5.9%) isolates for TMP-SMX, 3/34 (8.8%) for levofloxacin and 4/34 (11.8%) for minocycline after retrospective application of the current CLSI minocycline breakpoint.

Levofloxacin resistance was observed in 3/34 isolates (8.8%) and TMP-SMX resistance in 2/34 isolates (5.9%) (**Figure 1**). Retrospective reassessment of the archived minocycline zone records using the current CLSI breakpoint identified 4/34 isolates (11.8%) as minocycline-resistant: S05 (12 mm), S08 (20 mm), S25 (20 mm) and S26 (20 mm). Among the remaining minocycline records, six exact zone diameters fell in the intermediate range (21–25 mm) and 13 in the susceptible range (≥26 mm); for 11 isolates the contemporaneous worksheet recorded only “>20 mm,” establis*hin*g non-resistance but not allowing retrospective distinction between intermediate and susceptible categories. S05 and S08 were resistant to all three tested agents after current-breakpoint reassessment. S25 was resistant to levofloxacin and minocycline but remained susceptible to TMP-SMX, whereas S26 was minocycline-resistant only. The archived zone records and revised minocycline classifications are detailed in **Supplementary Table 4**.

### Distribution of selected resistance-associated determinants

**Figure 2:**
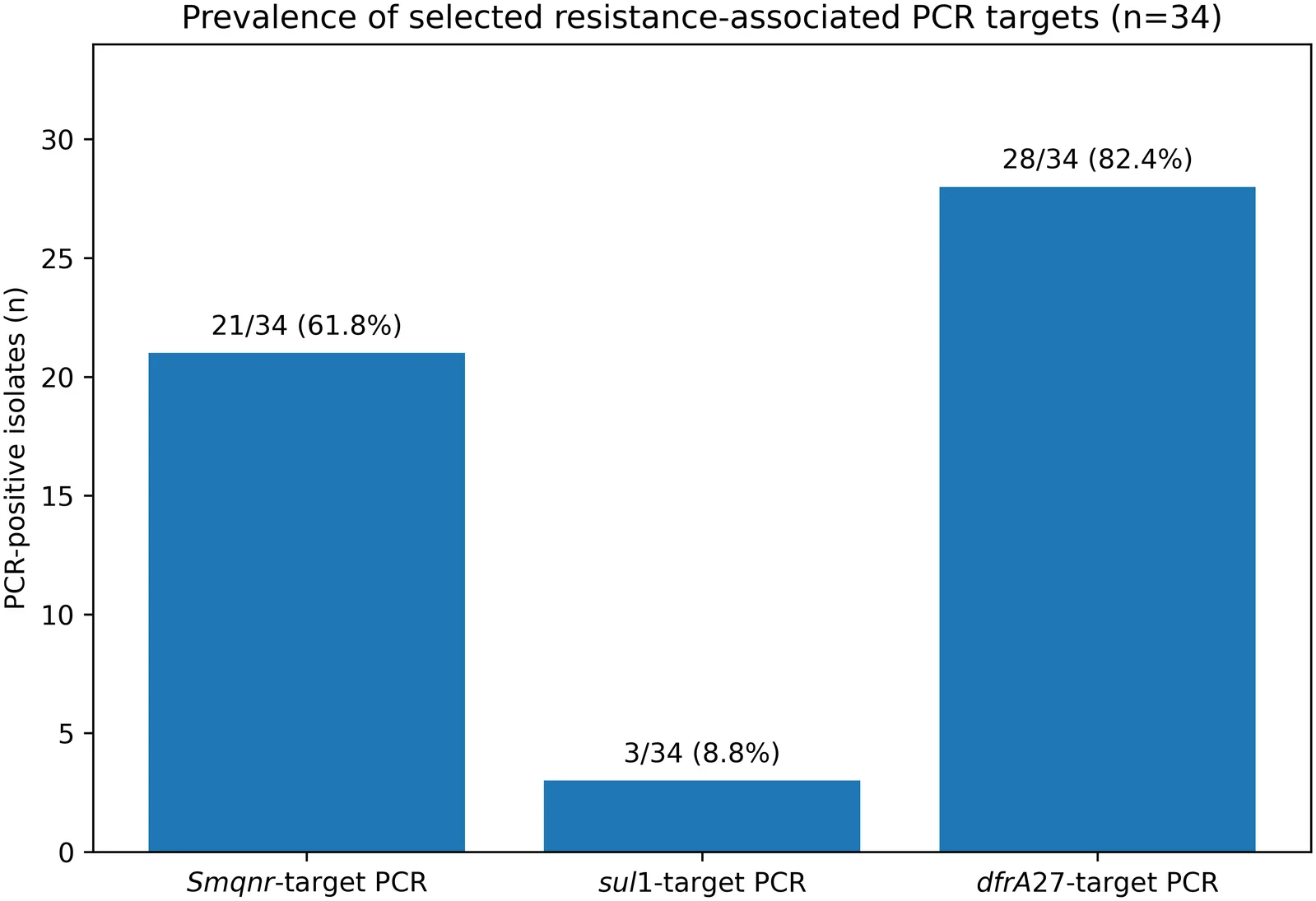
Prevalence of selected resistance-associated PCR targets among 34 clinical-culture *Stenotrophomonas maltophilia* isolates. Bars indicate the number and percentage of isolates with *Smqnr*-, *sul1*- and *dfrA27*-target PCR signals. The *dfrA27*-target result was not independently sequence-confirmed and should not be interpreted as confirmed *dfrA27* carriage; the same target-PCR principle applies to unvalidated PCR-only signals.

**Figure 3:**
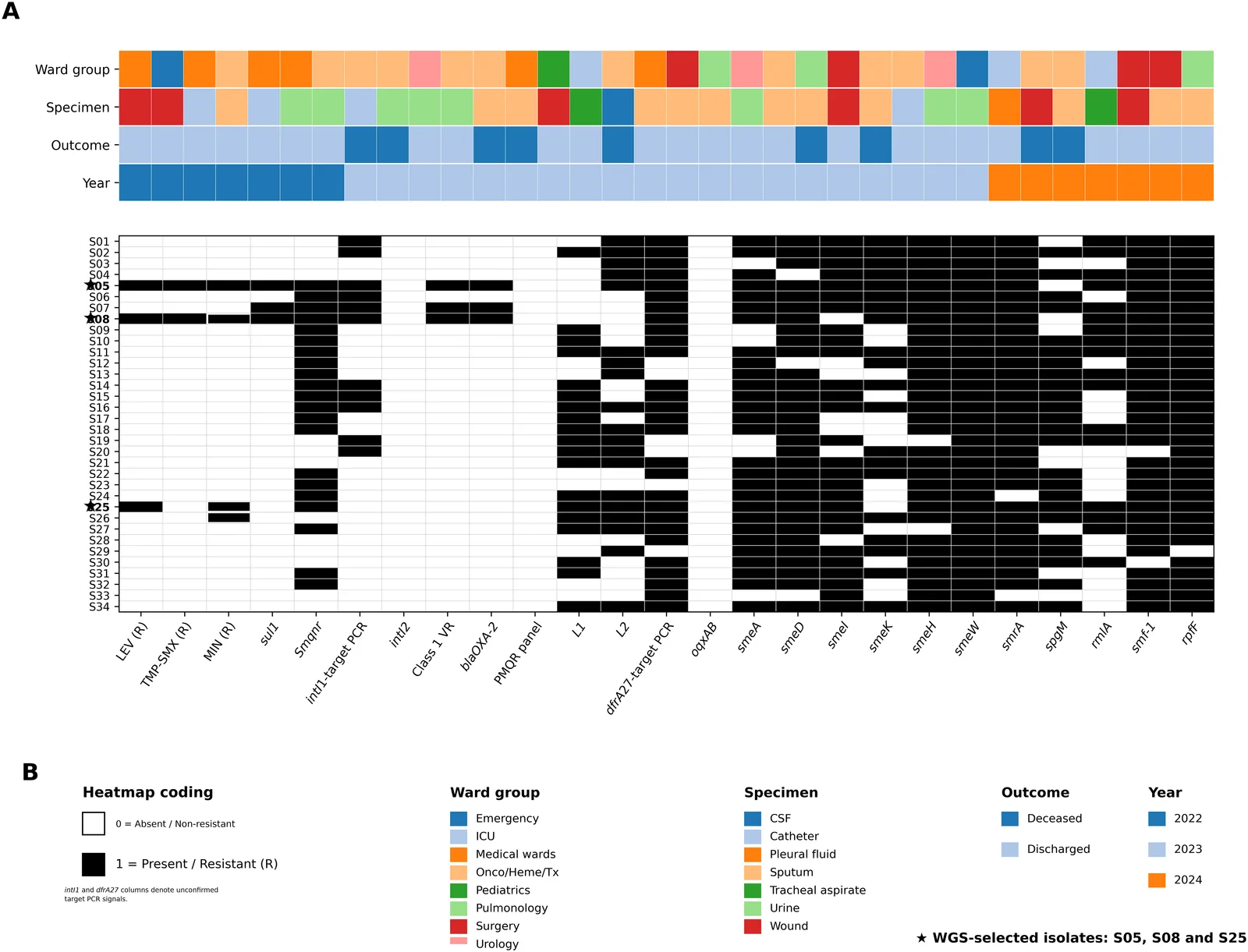
Integrated heatmap of specimen characteristics, antimicrobial resistance phenotypes and selected molecular markers among 34 clinical-culture *Stenotrophomonas maltophilia* isolates. **(A)** The enlarged heatmap shows individual isolates, resistance phenotypes and PCR markers; black cells indicate resistance or target-PCR positivity, and white cells indicate non-resistance or target negativity. Minocycline resistance reflects retrospective application of the current CLSI breakpoint. *intI1* and *dfrA27* columns denote unconfirmed target PCR signals. **(B)** Coding keys show ward group, specimen type, clinical outcome and year of isolation. WGS-selected isolates S05, S08 and S25 are indicated.

The distribution of selected PCR targets is summarized in **Figure 2** and detailed for each isolate in **Supplementary Table 4**. *Smqnr*-target PCR positivity was observed in 21/34 isolates (61.8%), *sul1*-target PCR positivity in 3/34 isolates (8.8%) and a *dfrA27*-target PCR signal/amplicon in 28/34 isolates (82.4%). *sul2* was not detected. The three *sul1*-target PCR-positive isolates were S05, S07 and S08. *intI1*-target PCR positivity was observed in 11/34 isolates. An approximately 2000-bp class 1 variable-region amplicon was detected in S05, S07 and S08 and was independently Sanger-sequenced as *blaOXA-2*-containing; this amplicon alone does not establish complete class 1 integron architecture. Because the *intI1*- and *dfrA27*-target amplicons were not independently sequence-confirmed, these results are reported as unconfirmed target-specific PCR signals rather than confirmed gene carriage. Other transferable quinolone resistance determinants included in the predefined PCR panel, namely *qnrA*, *qnrB*, *qnrC*, *qnrD*, *qnrS*, *aac(6′)-Ib-cr* and *qepA*, were not detected. Similarly, *oqxA* and *oqxB* were not detected. *L1* and/or *L2* β-lactamase targets were detected in 26/34 isolates. Efflux-associated markers were widely distributed: *smeW* was detected in all isolates, followed by *smeH* and *smrA* in 32/34 isolates, *smeD* in 31/34, *smeA* in 28/34, *smeI* in 27/34 and *smeK* in 20/34. Biofilm-associated markers were also frequent, with *rpfF* detected in 33/34 isolates, *smf-1* in 32/34, *spgM* in 24/34 and *rmlA* in 17/34. The integrated distribution of specimen variables, antimicrobial resistance phenotypes and molecular markers is shown in **Figure 3**.

### Phenotype–genotype associations

Associations between selected phenotypic resistance patterns and molecular targets are shown in **Table 1** and are considered exploratory because of the sparse resistant groups. Among the three *sul1*-target PCR-positive isolates, two were resistant to TMP-SMX, whereas only one of the 32 TMP-SMX-susceptible isolates was *sul1*-target PCR positive (Fisher’s exact p = 0.005; Haldane–Anscombe-corrected OR 105.0, 95% CI 3.33–3307.71). The *dfrA27*-target PCR signal was widely distributed among both TMP-SMX-resistant and TMP-SMX-susceptible isolates and showed no evidence of association (p = 1.000; corrected OR 1.23, 95% CI 0.05–28.77). All three levofloxacin-resistant isolates were *Smqnr*-target PCR positive, but this target was also frequent among levofloxacin-susceptible isolates (p = 0.270; corrected OR 5.11, 95% CI 0.24–107.34). The very wide confidence intervals emphasize the imprecision of these sparse-data effect estimates.

**Table 1:** Exploratory phenotype–genotype associations of selected resistance determinants among 34 clinical-culture *Stenotrophomonas maltophilia* isolates.

Comparison	Resistant +, n/N (%)	Susceptible +, n/N (%)	Fisher p	Corrected OR (95% CI)	Interpretation
TMP-SMX resistance vs *sul1*-target PCR positivity	2/2 (100%)	1/32 (3.1%)	0.005	105.0 (3.33–3307.71)	Exploratory association; very imprecise
TMP-SMX resistance vs *dfrA27*-target PCR positivity	2/2 (100%)	26/32 (81.3%)	1.000	1.23 (0.05–28.77)	No evidence of association
Levofloxacin resistance vs *Smqnr*-target PCR positivity	3/3 (100%)	18/31 (58.1%)	0.270	5.11 (0.24–107.34)	No evidence of association; very imprecise

TMP-SMX, trimethoprim-sulfamethoxazole; OR, odds ratio; CI, confidence interval. Two-sided Fisher’s exact tests were considered exploratory because only two isolates were TMP-SMX-resistant and three were levofloxacin-resistant. Because each 2 × 2 table contained a zero cell, Haldane–Anscombe-corrected ORs and approximate 95% CIs were calculated after adding 0.5 to all four cells. QRDR findings are described separately in the text because no hotspot mutation was detected in any isolate.

### Sequence analysis of quinolone resistance-associated genes

Sequence analysis of the quinolone resistance-associated genes *gyrA*, *gyrB*, *parC* and *parE* did not identify hotspot quinolone resistance-determining region (QRDR) mutations in any of the 34 isolates. All analyzed sequences were interpreted as wild-type or as carrying only non-QRDR polymorphisms relative to the *S. maltophilia* K279a reference genome within the analyzed regions. Therefore, QRDR hotspot mutations did not explain the levofloxacin-resistant phenotype observed in S05, S08 and S25. The complete QRDR sequence interpretation is provided in **Supplementary Table 3**.

### PFGE-based genetic relatedness

**Figure 4:**
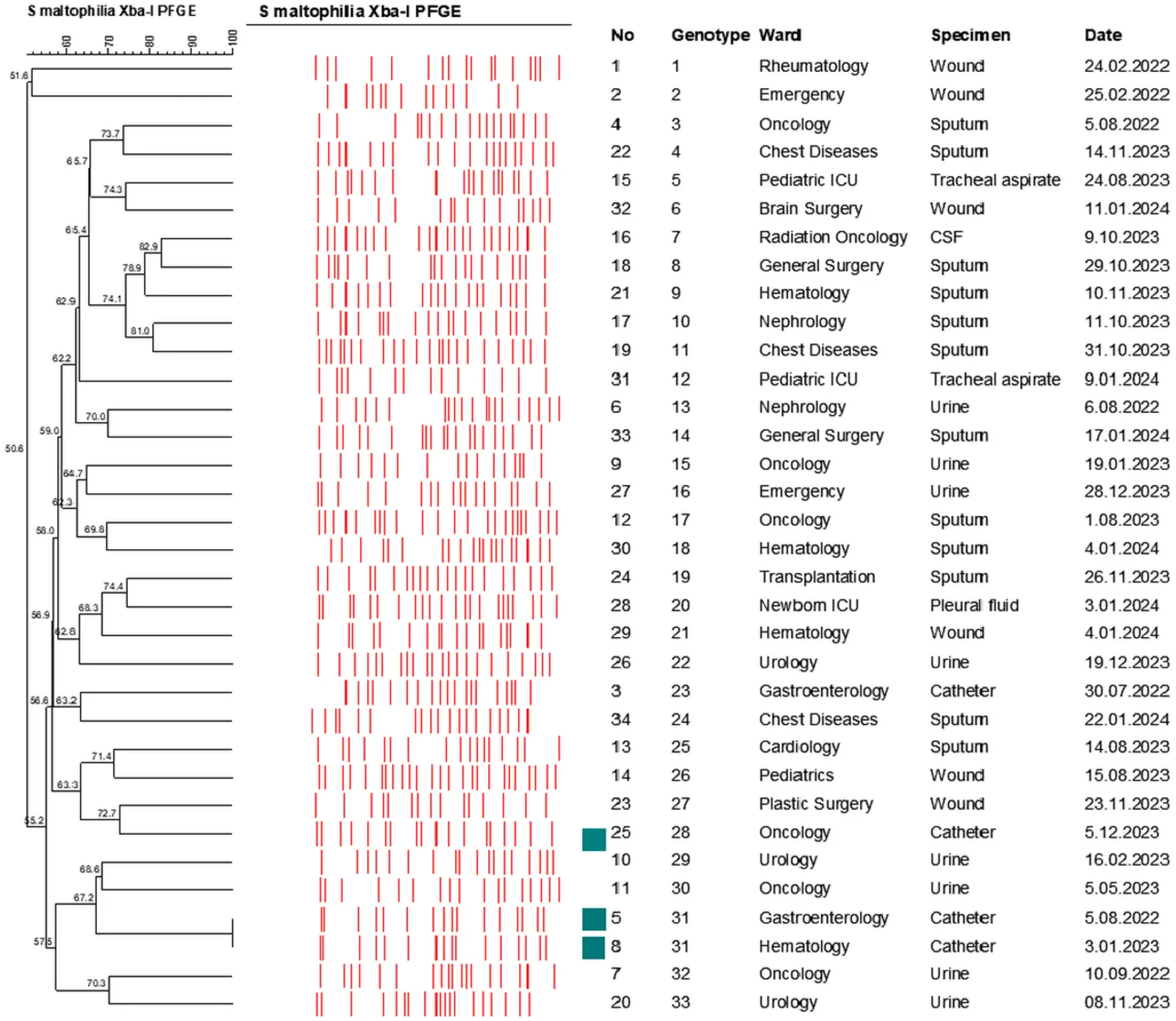
*Xba*I-PFGE dendrogram of clinical-culture *Stenotrophomonas maltophilia* isolates and associated specimen metadata. The dendrogram illustrates PFGE-based genetic relatedness among 34 isolates. Band-position tolerance and optimization were both 1.0%, and PFGE genotypes were assigned using an 85% similarity cutoff. Overall, 33 PFGE genotypes were identified; only S05 and S08 shared the same PFGE genotype (genotype 31), whereas S25 belonged to a distinct PFGE genotype. The accompanying metadata include isolate number, PFGE genotype, hospital ward, specimen type and date of isolation.

*Xba*I-PFGE revealed substantial genetic diversity among the 34 clinical-culture *S. maltophilia* isolates (**Figure 4**). Overall, 33 PFGE genotypes were identified, and no PFGE genotype predominated in the collection. Only two isolates, S05 and S08, shared the same PFGE genotype (genotype 31) at the predefined 85% similarity cutoff, corresponding to 5.9% (2/34) of the collection. Both isolates were resistant to TMP-SMX. In contrast, S25, which was selected as a comparative WGS isolate, belonged to a distinct PFGE genotype. The representative PFGE gel image supporting the dendrogram is provided in **Supplementary Figure 1**.

### Whole-genome sequencing of selected isolates

Three representative isolates, S05, S08 and S25, were selected for WGS. S05 and S08 were selected because both were TMP-SMX-resistant and shared the same PFGE genotype, whereas S25 was included as a comparative isolate representing a distinct phenotypic and PFGE background. The WGS-selected isolates are indicated in **Figures 3**, **4**. The draft assemblies contained 25–31 contigs, total assembly sizes of approximately 4.36–4.65 Mb, N50 values of approximately 315–317 kb and GC contents of 66.5–66.8%. BUSCO analysis showed complete recovery of 124/124 conserved single-copy orthologs in S05 and S08, and 122/124 complete orthologs in S25. Raw theoretical depths were approximately 697×, 777× and 841× for S05, S08 and S25, respectively, whereas the randomly subsampled assembly-input datasets yielded approximately 149× depth for each assembly. Assembly, QC and annotation metrics are summarized in **Supplementary Table 5**. S05 and S08 shared resistance-associated features not observed in S25, including a putative Sul-family/*folP*-like DHPS annotation, a mercury-resistance module and two assembly-predicted hits each of *bla_OXA-2_* and *ant(3′′)*. No additional acquired sul determinant was identified in the available WGS annotation outputs. Common intrinsic or background determinants, including *L1* and multiple efflux-associated annotations, were detected across the selected genomes. The complete WGS-based summary is provided in **Supplementary Table 5**.

### Genomic context of the shared resistance island in S05 and S08

**Figure 5:**
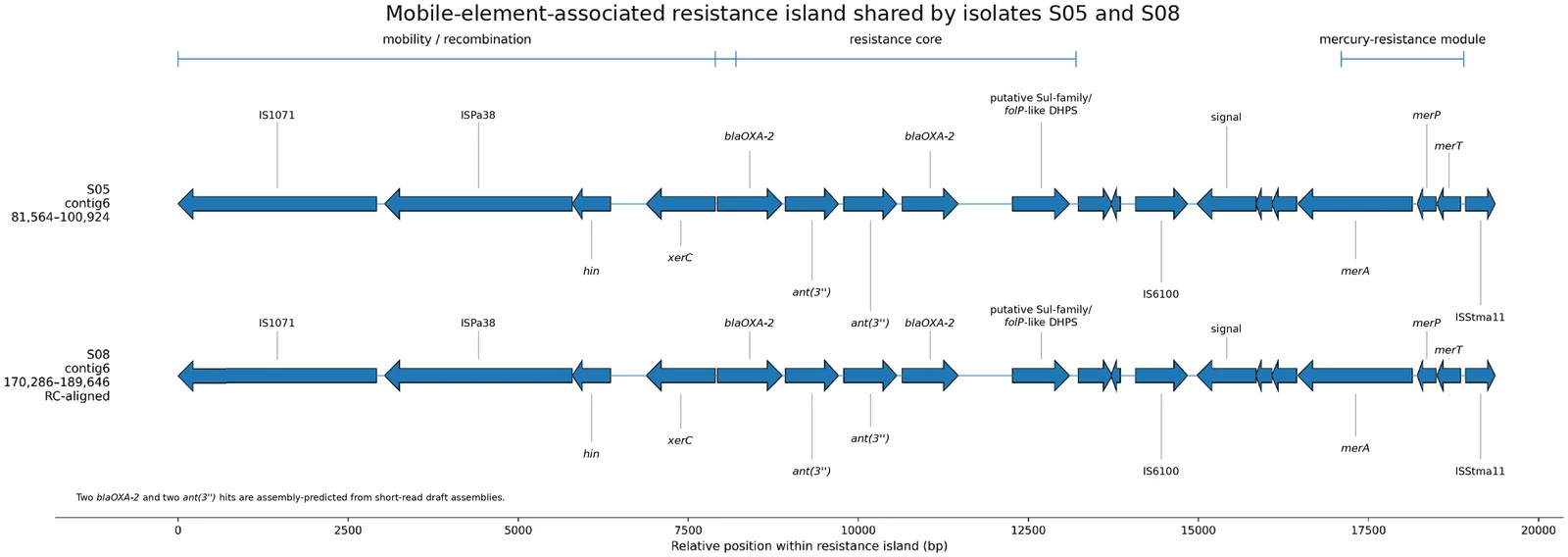
Gene-neighborhood organization of the mobile-element-associated resistance region identified in the WGS-selected isolates S05 and S08, which shared the same PFGE genotype. The 19,361-bp region from contig 6 is shown, with the S08 sequence reverse-complemented for alignment with S05. Pairwise nucleotide identity across the analyzed segment was 100%. The region contains a putative Sul-family/*folP*-like dihydropteroate synthase (DHPS) annotation, two assembly-predicted hits each of *bla_OXA-2_* and *ant(3’’)*, mobility-associated elements and a mercury-resistance module. Copy number and replicon location were not independently confirmed. The identical region wit*hin* one PFGE genotype does not demonstrate horizontal transfer.

Gene-neighborhood analysis identified an identical 19,361-bp mobile-element-associated resistance region in S05 and S08 (**Figure 5**). In S05, the region was located on contig 6 at positions 81,564–100,924; in S08, the corresponding region was located on contig 6 at positions 170,286–189,646 and was reverse-complemented for alignment. The two regions displayed 100% nucleotide identity across the analyzed segment. The region contained *IS1071*, *ISPa38*, *hin*, *xerC*, *IS6100* and *ISStma11*, together with the putative Sul-family/*folP*-like DHPS annotation, two assembly-predicted hits each of *blaOXA-2* and *ant(3′′)*, and a mercury-resistance module including *merA*, *merP* and *merT*. The corresponding region was absent from S25. For the DHPS locus, ABRicate/MEGARes reported SULI accession MEG_6615 at 99.90% identity and 100% coverage; Prokka annotated the corresponding 840-nt CDS as *folP_1*/dihydropteroate synthase. Coordinates, sequence and thresholds are provided in **Supplementary Table 5**. Copy number and replicon location were not independently validated by locus-specific coverage/junction analysis or long-read sequencing. Although PCR detected *intI1*-target positivity and a *blaOXA-2*-containing variable-region amplicon in S05 and S08, *intI1* was not directly confirmed in the WGS annotation outputs, and the variable-region amplicon alone does not establish complete class 1 integron architecture. The region was therefore interpreted as a mobile-element-associated resistance island rather than a confirmed class 1 integron. Because S05 and S08 shared the same PFGE genotype, the identical region was not interpreted as evidence of horizontal transfer; shared ancestry, vertical inheritance or persistence wit*hin* an island-carrying PFGE-defined genotype remain compatible explanations.

## Discussion

In this single-center collection of 34 clinical-culture *Stenotrophomonas maltophilia* isolates, phenotypic resistance to the tested agents remained limited, whereas selected resistance-, efflux- and biofilm-associated PCR targets were widely distributed. Resistance rates were 5.9% for TMP-SMX and 8.8% for levofloxacin; minocycline resistance was 11.8% after retrospective reassessment using current CLSI breakpoints. In contrast, *Smqnr*- and *dfrA27*-target PCR positivity, multiple *sme*/*smrA* markers and several biofilm-related markers were frequent. This discordance indicates that target detection alone is not necessarily predictive of phenotypic resistance and that PCR-only findings require interpretation in relation to genomic context, phenotypic susceptibility and, where possible, orthogonal sequence confirmation.

TMP-SMX, levofloxacin and minocycline remain among the main agents considered for *S. maltophilia* infections, although their relative roles continue to evolve as new clinical guidance and susceptibility data become available. In the present series, resistance to TMP-SMX and levofloxacin was lower than commonly reported in many surveillance datasets. Retrospective application of the revised CLSI minocycline breakpoint increased the resistance count from the original one isolate to four isolates (11.8%), illustrating the importance of reporting the breakpoint standard used. A recent systematic review and meta-analysis reported that resistance rates vary substantially across regions and settings, with minocycline generally showing among the lowest pooled resistance rates (Banar et al., 2023). Similarly, a large contemporary U.S. surveillance study of 1,400 clinical *S. maltophilia* isolates collected from 2019 to 2023 reported high susceptibility to TMP-SMX, moderate-to-high susceptibility to minocycline and lower susceptibility to levofloxacin, supporting the need for isolate-specific susceptibility testing, particularly when levofloxacin is considered (Sader et al., 2025).

Current guidance also reflects the increasing clinical complexity of *S. maltophilia*. The 2024 IDSA guidance includes *S. maltophilia* among antimicrobial-resistant Gram-negative pathogens for which treatment recommendations are provided, and EUCAST updated its dedicated *S. maltophilia* guidance in November 2024 (European Committee on Antimicrobial Susceptibility Testing, 2024; Tamma et al., 2024). CLSI revised *S. maltophilia* breakpoints in M100 Ed34, including lower minocycline breakpoints, and subsequent editions retained these criteria while also including cautionary comments regarding monotherapy for some agents (Clinical and Laboratory Standards Institute, 2025; Clinical and Laboratory Standards Institute, 2026). Although the present study was not designed to evaluate treatment outcomes or newer agents, the local TMP-SMX, levofloxacin and minocycline resistance rates remained limited. Nevertheless, the presence of a multideterminant mobile-element-associated region in selected isolates indicates that ongoing surveillance remains necessary.

TMP-SMX resistance was detected only in S05 and S08. Both resistant isolates were *sul1*-target PCR positive, whereas *sul1*-target PCR positivity was found in only one TMP-SMX-susceptible isolate; this exploratory comparison yielded Fisher’s exact p = 0.005, but the corrected OR had a very wide 95% confidence interval because only two resistant isolates were available. This pattern is consistent with previous reports linking *sul1*, often in class 1 integron-associated contexts, to TMP-SMX resistance in *S. maltophilia* (Toleman et al., 2007; Hu et al., 2011; Ozkaya et al., 2014; Chung et al., 2015; Hu et al., 2016). However, the *sul1* PCR product in the present study was not independently sequenced, and should not be treated as sequence confirmation of the WGS DHPS locus. *sul2* was not detected. Because *sul3* and *sul4* were not included in the original targeted protocol, the possible contribution of other sul variants cannot be excluded.

By contrast, a *dfrA27*-target PCR signal/amplicon was observed in 82.4% of isolates but did not distinguish TMP-SMX-resistant from susceptible isolates (exploratory Fisher’s exact p = 1.000) and was not directly confirmed in any of the three WGS annotation outputs. This pattern raises concern for non-specific amplification or cross-reactivity with a related dihydrofolate-reductase sequence. Without amplicon sequencing, however, the result cannot be classified definitively as false positivity. We therefore report it as an unconfirmed *dfrA27*-target PCR signal and do not interpret it as confirmed *dfrA27* carriage or as a predictor of TMP-SMX resistance.

The presence of sul genes on mobile genetic elements in *S. maltophilia* has been established previously (Hu et al., 2011; Ozkaya et al., 2014; Chung et al., 2015; Hu et al., 2016). Accordingly, the novelty of the present study does not lie in the first detection of a mobile *sul* determinant. Its contribution is the integration of phenotypic susceptibility, targeted PCR/Sanger results, PFGE and short-read WGS wit*hin* a single local clinical-culture collection; the characterization of an identical 19,361-bp multideterminant region in two TMP-SMX-resistant isolates sharing the same PFGE genotype; and the demonstration that widespread *dfrA27*-target PCR positivity was discordant with both phenotype and the selected WGS results. Because systematic comparison with public genomes was not performed, we do not claim that this region represents a previously undescribed island, a distinct lineage-associated structure or evidence of global distribution.

### Fluoroquinolone resistance: *Smqnr*, absence of QRDR hotspot mutations and the likely role of efflux regulation

Levofloxacin resistance was detected in three isolates, S05, S08 and S25. All three levofloxacin-resistant isolates were *Smqnr*-target PCR positive, but *Smqnr*-target PCR positivity was also frequent among levofloxacin-susceptible isolates, and the exploratory association was not statistically significant. This result is biologically plausible. *Smqnr* is a chromosome-carried Qnr-like determinant first described in *S. maltophilia* and has been shown to reduce susceptibility to fluoroquinolones, but its presence alone does not necessarily predict clinical resistance (Shimizu et al., 2008; Sánchez and Martínez, 2010).

No additional transferable quinolone resistance determinants in the predefined panel were detected, and sequence analysis of *gyrA*, *gyrB*, *parC* and *parE* did not reveal QRDR hotspot mutations. Thus, levofloxacin resistance was not explained by QRDR hotspot mutations, *Smqnr* alone or the tested transferable determinants. Other mechanisms were not assessed by the targeted panel. In particular, overexpression of the major-facilitator transporter *mfsA* and mutations affecting its redox-responsive regulator *soxR* have been associated with increased fluoroquinolone resistance in *S. maltophilia* (Vattanaviboon et al., 2018; Kanawong et al., 2026). These and other regulatory or permeability mechanisms remain plausible but untested explanations in the present isolates.

Multiple efflux-associated PCR markers, including *smeA*, *smeD*, *smeK*, *smeI*, *smeH*, *smeW* and *smrA*, were widely distributed. These markers were selected on the basis of previously reported associations and served as targeted screening loci; their presence does not establish that the corresponding operons were complete, functional or overexpressed. Quantitative expression analysis was not part of the predefined retrospective study design and would require standardized growth and exposure conditions, biological replicates and an adequately powered set of resistant isolates. We therefore avoid attributing resistance to efflux overexpression and identify qRT-PCR or transcriptomic analysis as a necessary component of future functional studies.

*intI1*-target PCR positivity was observed in 11 isolates, and an approximately 2000-bp class 1 variable-region amplicon was detected and Sanger-sequenced as *bla_OXA-2_*-containing in S05, S07 and S08. This distinction between unconfirmed PCR-only signals and independently sequence-supported amplicon content is important. Although class 1 integrons are recognized platforms for resistance gene cassettes and previous studies have linked integrons, *sul* genes and *dfrA* genes to TMP-SMX resistance (Hu et al., 2011; Ozkaya et al., 2014; Chung et al., 2015; Hu et al., 2016; García et al., 2023), the *bla_OXA-2_*-containing variable-region amplicon alone does not establish complete class 1 integron architecture, and the present PCR results do not establish collection-wide integron architecture or functionality.

The WGS findings further limit the interpretation. *intI1* was not directly confirmed in the annotation outputs of S05, S08 or S25, and the S05/S08 region could not be assigned definitively to a plasmid or chromosome from short-read draft assemblies. Its insertion sequences and recombination-associated features are compatible with mobility potential, but they do not demonstrate transfer. Because S05 and S08 shared the same PFGE genotype, the identical region is compatible with shared ancestry, vertical inheritance or persistence of an island-carrying PFGE-defined genotype; PFGE alone does not establish whole-genome clonality. S07, the *sul1*-target PCR-positive isolate from a different PFGE background, was not sequenced; therefore, sharing of the region across distinct PFGE genotypes cannot be evaluated in this study.

The two *blaOXA-2* and *ant(3′′)* hits in each S05/S08 draft assembly are described as assembly-predicted rather than confirmed copy numbers. Short-read assembly can misresolve repeated regions, and definitive copy-number confirmation would require locus-specific read-depth/junction analysis or long-read sequencing. The co-location of *merA*, *merP* and *merT* with antimicrobial-resistance determinants raises a theoretical possibility of co-selection under relevant environmental metal exposure. However, mercury exposure and functional effects were not measured, and the present data do not show that the mercury-resistance module contributes to maintenance of the island or directly mediates antibiotic resistance.

PFGE demonstrated marked genetic diversity, with 33 genotypes among 34 isolates and no genotype predominating in the collection. These data describe heterogeneity of XbaI macrorestriction profiles; PFGE alone cannot establish or exclude patient-to-patient transmission, and no outbreak or transmission inference was made from the typing data.

The combined PFGE/WGS findings show that S05 and S08 shared the same PFGE genotype and carried an identical resistance region, but they do not establish whole-genome clonality or horizontal transfer between the isolates. S25 provided a useful local comparator because it belonged to a distinct PFGE genotype and lacked the S05/S08 region. After retrospective application of the current minocycline breakpoint, both S05 and S08 were minocycline-resistant; therefore, the previously noted phenotypic difference between these isolates is no longer supported under the current interpretation.

Biofilm-related genes were frequent in this collection, particularly *rpfF*, *smf-1*, *spgM* and *rmlA*. Previous studies have associated these genes with biofilm formation, adherence, surface persistence and chronic colonization potential in *S. maltophilia* (Zhuo et al., 2014). In the present study, these findings should be interpreted as evidence of genetic potential rather than direct phenotypic biofilm production, because quantitative biofilm assays were not performed.

The isolates originated from several specimen types, but the study was not designed to test site-specific genomic adaptation. Small and uneven specimen-site groups, the availability of WGS for only three isolates, and incomplete distinction between colonization and infection preclude meaningful inference regarding relationships among specimen site, genotype and phenotype. These data are therefore presented descriptively rather than as evidence of niche-specific adaptation.

From a clinical perspective, most isolates did not meet resistance criteria for TMP-SMX, levofloxacin or minocycline. However, interpretation should remain local and susceptibility-based, and the retrospective minocycline reassessment also illustrates the effect of breakpoint revisions on categorical results. Current guidance emphasizes the complexity of *S. maltophilia* therapy, the limited clinical-outcome evidence for several agents and the need to avoid over-reliance on single molecular markers or historical assumptions (European Committee on Antimicrobial Susceptibility Testing, 2024; Tamma et al., 2024; Sader et al., 2025; Clinical and Laboratory Standards Institute, 2026).

From an infection-control perspective, the high PFGE diversity and absence of a predominant genotype indicate a heterogeneous collection. S05 and S08 shared the same PFGE genotype, but PFGE does not establish direction or occurrence of transmission, and no patient-to-patient transmission or outbreak inference was made. Surveillance should therefore integrate phenotypic susceptibility, molecular typing and cautious interpretation of resistance-determinant screening, particularly in high-risk hospital settings.

This study has several limitations. First, it was a single-center study with a modest number of isolates and only three levofloxacin-resistant and two TMP-SMX-resistant isolates; the Fisher-test results and corresponding sparse-data effect sizes are therefore exploratory and imprecise. Second, the predefined PCR panel was hypothesis-driven and did not constitute a whole-collection resistome, regulatory-network or operon-function analysis. *intI1*- and *dfrA27*-target PCR products were not independently sequence-confirmed, and their absence from the three WGS annotation outputs raises the possibility of primer cross-reactivity or amplification of related sequences. The *blaOXA-2*-containing class 1 variable-region amplicon does not by itself establish complete class 1 integron architecture. Third, WGS was performed only for S05, S08 and S25 as a supporting genomic-context analysis. S07 was not sequenced, public-genome phylogenomics was not performed, and the origin, lineage distribution and sharing of the resistance region across distinct PFGE genotypes cannot be determined. Fourth, short-read assemblies did not allow definitive replicon assignment or independent validation of the assembly-predicted *blaOXA-2* and *ant(3′′)* copy numbers. Fifth, gene-expression and regulatory analyses were not performed; therefore, the potential roles of efflux overexpression and *mfsA*/*soxR*-associated mechanisms remain unresolved. Sixth, biofilm-associated markers were assessed genotypically without phenotypic biofilm assays. Seventh, although the final minocycline analysis was based on the QC-acceptable repeat run, 11 repeat-run worksheet entries were retained only as “>20 mm”; these isolates do not meet the current resistance breakpoint, but retrospective separation of intermediate from susceptible categories is not possible. Finally, the heterogeneous specimen sites, limited WGS sampling and incomplete colonization-versus-infection classification precluded site-specific genomic or clinical-outcome analysis.

In conclusion, clinical-culture *S. maltophilia* isolates from this tertiary-care hospital showed limited phenotypic resistance to TMP-SMX, levofloxacin and minocycline, although retrospective application of current CLSI minocycline breakpoints increased the minocycline-resistant proportion to 11.8%. TMP-SMX resistance showed an exploratory association with *sul1*-target PCR positivity, but the *sul1* PCR product itself was not sequence-validated. Short-read WGS identified the same 19,361-bp mobile-element-associated resistance region, including a putative Sul-family/*folP*-like DHPS annotation, in the two TMP-SMX-resistant isolates S05 and S08 sharing the same PFGE genotype. This finding is compatible with shared ancestry, vertical inheritance or persistence of an island-carrying PFGE-defined genotype; horizontal transfer was not demonstrated. The discordance between *dfrA27*-target PCR positivity, phenotype and WGS underscores the need to combine molecular screening with phenotypic susceptibility and orthogonal confirmation. The genomic component should be interpreted as a targeted local context analysis rather than evidence of cross-genotype or global dissemination.

## Data Availability

The raw whole-genome sequencing reads generated in this study are publicly available in the NCBI Sequence Read Archive under BioProject PRJNA1481107. The isolate-to-record mapping is as follows: S05, BioSample SAMN61058391 and SRA run SRR39278206; S08, BioSample SAMN61058392 and SRA run SRR39278205; and S25, BioSample SAMN61058393 and SRA run SRR39278204. The BioProject and associated records were publicly released on 17 August 2026.
