# Supplementary material for: Genomic characterization of a mobile-element-associated resistance island in trimethoprim-sulfamethoxazole-resistant Stenotrophomonas maltophilia isolates sharing the same PFGE genotype
Source: Front Cell Infect Microbiol. 2026 Sep 11;16:1926102. doi: 10.3389/fcimb.2026.1926102 (PMC13612193; doi:10.3389/fcimb.2026.1926102)
Supplement: Supplementary file 1 [file Table1.docx]

**Supplementary Table S1**

Properties of the oligonucleotide primers used in the study

| Group of Primer | Target Gene | Sequence (5’ → 3’) | Product Size (bp) | References |
| --- | --- | --- | --- | --- |
| Integrons | *IntI1* integrase | GGTCAAGGATCTGGATTTGG  ACATGCGTGTAAATCATCGTC | 500 | [13] |
|  | *IntI2* integrase | CACGGATATGCGACAAAAAGGT  GTAGCAAACGAGTGACGAAATG | 740 | [13] |
|  | *IntI3* integrase | AGTGGGTGGCGAATGAGTG  TGTTCTTGTATCGGCAGGTG | 600 | [14] |
|  | Class 1 integron | GGCATCCAAGCAGCAAG  AAGCAGACTTGACCTGA | Variable | [15] |
|  | Class 2 integron | GATGCCATCGCAAGTACGAG  CGGGATCCCGGACGGATGCACGATTTGTA | Variable | [16] |
| Sulphonamides | *Sul1* | CTTCGATGAGAGCCGGCGGC  GCAAGGCGGAAACCCGCGCC | 238 | [17] |
|  | *Sul2* | GCGCTCAAGGCAGATGGCATT  GCGTTTGATACCGGCACCCGT | 793 | [17] |
| Βeta Lactamases | *L1* | CACACCTGGCAGATCGGCAC  GCCGCATCCGCGTAGGC | 459 | [18] |
|  | *L2* | CGATTCCTGC AGTTCAGT  CGGTTACCTC ATCCGATC | 870 | [18] |
| Dihydrofolate Reductases | *dfrA1* | CTTGTTAACCCTTTTGCCAGA  TTGTGAAACTATCACTAATGGTAG |  | [3] |
|  | *dfrA5* | ATCGTCGATATATGGAGCGTA  TCCACACATACCCTGGTCCG |  | [3] |
|  | *dfrA12* | ATGAACTCGGAATCAGTACGC  TTAGCCGTTTCGACGCGCAT |  | [3] |
|  | *dfrA13* | CTCATCTGCTGGCTATCTCA  GAAACTATCACTAATGGCAGC |  | [3] |
|  | *dfrA17* | GTTAGCCTTTTTTCCAAATCTGGTATG  TTGAAAATATTATTGATTTCTGCAGTG |  | [3] |
|  | *dfrA27* | AAGAGTCTGATCGCCCATGCCG  TAAAGCAATAACTTACAATC |  | [3] |
| Quinolones | *gyrA* | AACTCAACGCGCACAGCAACAAGCC  CCAGTTCCTTTTCGTCGTAGTTGGG | 300 | [5] |
|  | *gyrB* | GATGACCCGCCGTAAGGGCG  GAAGGTCAGCAGCAGGGTGC | 369 | [5] |
|  | *parC* | ATCGGCGACGGCCTGAAGCC CGGGATTCGGTATAACGCAT | 273 | [5] |
|  | *parE* | CCGGCGCTGCCTGGCAAACTG  CGCCCAGGCCCTTGAAGCGGG | 519 | [5] |
|  | Sm*qnr* | ACACAGAACGGCTGGACTGC  TTCAACGACGTCGAGCTGT | 817 | [4] |
|  | *qnrA* | ATTTCTCACGCCAGGATTTG  GATCGGCAAAGGTTAGGTCA | 516 | [19] |
|  | *qnrB* | GATCGTGAAAGCCAGAAAGG  ACGATGCCTGGTAGTTGTCC | 469 | [19] |
|  | *qnrC* | GGGTTGTACATTTATTGAATCG  CACCTACCCATTTATTTTCA | 310 | [6] |
|  | *qnrD* | GGGTTGATTTAACTGATAC  TTCGCACTTTTCTAATATGAC | 310 | [6] |
|  | *qnrS* | GCAAGTTCATTGAACAGGGT  TCTAAACCGTCGAGTTCGGCG | 428 | [20] |
|  | *aac(6’)-Ib-cr* | TTGCGATGCTCTATGAGTGGCTA  CTCGAATGCCTGGCGTGTTT | 482 | [21] |
| Efflux Genes | *qepA* | AACTGCTTGAGCCCGTAGAT  GTCTACGCCATGGACCTCAC | 596 | [22] |
|  | *oqxA* | CTTGCACTTAGTTAAGCGCC  GAGGTTTTGATAGTGGAGGTAGG | 866 | [6] |
|  | *oqxB* | GCGGTGCTGTCGATTTTA  TACCGGAACCCATCTCGAT | 781 | [6] |
|  | *smeA* | AAGGCCATCGATGGCAAGGC  TCCGGGTTCGGAATGACCG | 146 | [2] |
|  | *smeD* | CCAAGAGCCTTTCCGTCAT TCTCGGACTTCAGCGTGAC | 150 | [2] |
|  | *smeK* | GACCTCGCAGACGCAGTCG CAGGTAGTCGCGCAGGGTC | 505 | [2] |
|  | *smeI* | TTCCGCGAAGGCCAGGAAGT  TCGTTCTGGCGCTTGGCTG | 107 | [2] |
|  | *smeW* | TTCGGCGACATCGTGCTCAA  CTTGAAGAAGCGGTTGAACGG | 843 | [2] |
|  | *smeH* | GTGGATGATCGGCTTCACGAT  CGCATAGCCCTGGTCTTCTT | 556 | [2] |
|  | *smrA* | GGTGTGGCCGGTGCTGCT  CGCGGTGCTTGACCGCCA | 677 | [2] |
| Biofilm Genes | *rmlA* | GCAAGGTCATCGACCTGG  TTGCCGTCGTAGAAGTACAGG | 82 | [23] |
|  | *rpfF* | CTGGTCGACATCGTGGTG  TGATCCGCATCATTTCATGC | 151 | [23] |
|  | *spgM* | GCTTCATCGAGGGCTACTACC  ATGCACGATCTTGCCGC | 80 | [23] |
|  | *smf-1* | GGAAGGTATGTCCGAGTCCGR  GCGGGTACGGCTACGATCAGTT | 674 | [7] |

**Supplementary Table S2**

Cycle parameters of polymerase chain reactions

| Reaction For Genes | First Denaturation | Denaturation | Elongation | Extension | Final Extension | Cycle |
| --- | --- | --- | --- | --- | --- | --- |
| *intI1/intI2* integrase | 94°C (5 min) | 94°C (1 min) | 57°C (5 min) | 72°C (1 min) | 72°C (10 min) | 34 |
| Class 1 Integron | 94°C (2 min) | 94°C (30 s) | 56°C (45 s) | 72°C (3 min) | 72°C (5min) | 34 |
| Class 2 Integron | 94°C (3 min) | 94°C (45 s) | 55°C (1 min) | 72°C (2 min) | 72°C (5 min) | 34 |
| *sul1/sul2* | 94°C (3 min) | 94°C (30 s) | 58°C (45 s) | 72°C (1 min) | 72°C (10 min) | 30 |
| Sm*qnr* | 94°C (5 min) | 94°C (45 s) | 59°C (45 s) | 72°C (45 s) | 72°C (5 min) | 36 |
| *qnrA/B/S/aac(6’)-Ib-cr/qepA* | 94°C (5 min) | 94°C (1 min) | 55°C (1 min) | 72°C (1 min) | 72°C (10 min) | 30 |
| *qnrC/D* | 95°C (5 min) | 95°C (30 s) | 56°C (30 s) | 72°C (1 min) | 72°C (7 min) | 30 |
| *gyrA/gyrB/parC/parE* | 94°C (5 min) | 94°C (1 min) | 54°C (1 min) | 72°C (1 min) | 72°C (5 min) | 30 |
| *dfrA* Genes | 95°C (5 min) | 95°C (1 min) | 55°C (1 min) | 72°C (2 min) | 72°C (10 min) | 35 |
| L1 | 94°C (3 min) | 94°C (30 s) | 65°C (30 s) | 72°C (30 s) | 72°C (5 min) | 35 |
| L2 | 94°C (3 min) | 94°C (30 s) | 55°C (30 s) | 72°C (30 s) | 72°C (5 min) | 35 |
| *oqxA/oqxB* | 95°C (5 min) | 95°C (30 s) | 65°C (30 s) | 72°C (1 min) | 72°C (7 min) | 30 |
| *smeA/D/K/I* | 95°C (4 min) | 95°C (30 s) | 59°C (30 s) | 72°C (1 min) | 72°C (5 min) | 25 |
| *smeW/H* | 95°C (4 min) | 95°C (30 s) | 58°C (30 s) | 72°C (1 min) | 72°C (5 min) | 25 |
| *smrA* | 95°C (4 min) | 95°C (30 s) | 63°C (30 s) | 72°C (1 min) | 72°C (5 min) | 25 |
| *smf-1* | 95°C (5 min) | 94°C (45 s) | 62°C (45 s) | 72°C (45 s) | 72°C (7 min) | 30 |
| *rmlA/spgM* | 95°C (5 min) | 94°C (20 s) | 60°C (20 s) | 72°C (30 s) | 72°C (10 min) | 30 |
| *rpfF* | 94°C (5 min) | 94°C (45 s) | 59°C (45 s) | 72°C (45 s) | 72°C (5 min) | 36 |

**Supplementary Table S3**

Sequence analysis of quinolone resistance-associated genes in *Stenotrophomonas maltophilia* isolates.

| Isolate | *gyrA* | *parC* | *gyrB* | *parE* | Interpretation |
| --- | --- | --- | --- | --- | --- |
| S01 | WT | WT | WT | WT | Wild-type or non-QRDR polymorphism |
| S02 | WT | WT | WT | WT | Wild-type or non-QRDR polymorphism |
| S03 | WT | WT | WT | WT | Wild-type or non-QRDR polymorphism |
| S04 | WT | WT | WT | WT | Wild-type or non-QRDR polymorphism |
| S05 | WT | WT | WT | WT | Wild-type or non-QRDR polymorphism |
| S06 | WT | WT | WT | WT | Wild-type or non-QRDR polymorphism |
| S07 | WT | WT | WT | WT | Wild-type or non-QRDR polymorphism |
| S08 | WT | WT | WT | WT | Wild-type or non-QRDR polymorphism |
| S09 | WT | WT | WT | WT | Wild-type or non-QRDR polymorphism |
| S10 | WT | WT | WT | WT | Wild-type or non-QRDR polymorphism |
| S11 | WT | WT | WT | WT | Wild-type or non-QRDR polymorphism |
| S12 | WT | WT | WT | WT | Wild-type or non-QRDR polymorphism |
| S13 | WT | WT | WT | WT | Wild-type or non-QRDR polymorphism |
| S14 | WT | WT | WT | WT | Wild-type or non-QRDR polymorphism |
| S15 | WT | WT | WT | WT | Wild-type or non-QRDR polymorphism |
| S16 | WT | WT | WT | WT | Wild-type or non-QRDR polymorphism |
| S17 | WT | WT | WT | WT | Wild-type or non-QRDR polymorphism |
| S18 | WT | WT | WT | WT | Wild-type or non-QRDR polymorphism |
| S19 | WT | WT | WT | WT | Wild-type or non-QRDR polymorphism |
| S20 | WT | WT | WT | WT | Wild-type or non-QRDR polymorphism |
| S21 | WT | WT | WT | WT | Wild-type or non-QRDR polymorphism |
| S22 | WT | WT | WT | WT | Wild-type or non-QRDR polymorphism |
| S23 | WT | WT | WT | WT | Wild-type or non-QRDR polymorphism |
| S24 | WT | WT | WT | WT | Wild-type or non-QRDR polymorphism |
| S25 | WT | WT | WT | WT | Wild-type or non-QRDR polymorphism |
| S26 | WT | WT | WT | WT | Wild-type or non-QRDR polymorphism |
| S27 | WT | WT | WT | WT | Wild-type or non-QRDR polymorphism |
| S28 | WT | WT | WT | WT | Wild-type or non-QRDR polymorphism |
| S29 | WT | WT | WT | WT | Wild-type or non-QRDR polymorphism |
| S30 | WT | WT | WT | WT | Wild-type or non-QRDR polymorphism |
| S31 | WT | WT | WT | WT | Wild-type or non-QRDR polymorphism |
| S32 | WT | WT | WT | WT | Wild-type or non-QRDR polymorphism |
| S33 | WT | WT | WT | WT | Wild-type or non-QRDR polymorphism |
| S34 | WT | WT | WT | WT | Wild-type or non-QRDR polymorphism |

**WT: sequence identical to the** Stenotrophomonas maltophilia **K279a reference genome (GenBank accession no. AM743169.1) within the analyzed region.**

**Supplementary Figure S1.**

Representative *Xba*I-PFGE gel image and dendrogram of the 34 clinical *Stenotrophomonas maltophilia* isolates. Isolates selected for whole-genome sequencing, S05, S08 and S25, are indicated with green boxes.
