# Supplementary material for: Genomic characterization of a mobile-element-associated resistance island in trimethoprim-sulfamethoxazole-resistant Stenotrophomonas maltophilia isolates sharing the same PFGE genotype
Source: Front Cell Infect Microbiol. 2026 Sep 11;16:1926102. doi: 10.3389/fcimb.2026.1926102 (PMC13612193; doi:10.3389/fcimb.2026.1926102)
Supplement: Supplementary file 2 [file Table4.docx]

**Table 1.** Exploratory phenotype–genotype associations of selected resistance determinants among 34 clinical-culture *Stenotrophomonas maltophilia* isolates

| **Comparison** | **Resistant +, n/N (%)** | **Susceptible +, n/N (%)** | **Fisher p** | **Corrected OR (95% CI)** | **Interpretation** |
| --- | --- | --- | --- | --- | --- |
| TMP-SMX resistance vs *sul1*-target PCR positivity | 2/2 (100%) | 1/32 (3.1%) | 0.005 | 105.0 (3.33–3307.71) | Exploratory association; very imprecise |
| TMP-SMX resistance vs *dfrA27*-target PCR positivity | 2/2 (100%) | 26/32 (81.3%) | 1.000 | 1.23 (0.05–28.77) | No evidence of association |
| Levofloxacin resistance vs *Smqnr*-target PCR positivity | 3/3 (100%) | 18/31 (58.1%) | 0.270 | 5.11 (0.24–107.34) | No evidence of association; very imprecise |

TMP-SMX, trimethoprim-sulfamethoxazole; OR, odds ratio; CI, confidence interval. Two-sided Fisher’s exact tests were considered exploratory because only two isolates were TMP-SMX-resistant and three were levofloxacin-resistant. Because each 2 × 2 table contained a zero cell, Haldane–Anscombe-corrected ORs and approximate 95% CIs were calculated after adding 0.5 to all four cells. QRDR findings are described separately in the text because no hotspot mutation was detected in any isolate.
